# What is the safest mode of delivery for extremely preterm cephalic/non-cephalic twin pairs? A systematic review and meta-analyses

**DOI:** 10.1186/s12884-017-1554-7

**Published:** 2017-11-29

**Authors:** Catherine Dagenais, Anne-Mary Lewis-Mikhael, Marinela Grabovac, Amit Mukerji, Sarah D. McDonald

**Affiliations:** 1 Department of Obstetrics & Gynecology, McMaster University, 1280 Main St W, HSC 3N52B, Hamilton, ON L8S 4K1 Canada; 2 Department of Health Research Methods, Evidence, and Impact, McMaster University, 1280 Main St W, Hamilton, ON L8S 4K1 Canada; 3 Department of Pediatrics, McMaster University, 1280 Main St W, Hamilton, ON L8S 4K1 Canada

**Keywords:** Twin, Extremely preterm, Extremely low birth weight, Vaginal delivery, Caesarean section, Breech presentation

## Abstract

**Background:**

Given the controversy around mode of delivery, our objective was to assess the evidence regarding the safest mode of delivery for actively resuscitated extremely preterm cephalic/non-cephalic twin pairs before 28 weeks of gestation.

**Methods:**

We searched Cochrane CENTRAL, MEDLINE, EMBASE and http://clinicaltrials.gov from January 1994 to January 2017. Two reviewers independently screened titles, abstracts and full text articles, extracted data and assessed risk of bias. We included randomized controlled trials and observational studies. Our primary outcome was a composite of neonatal death (<28 days of life) and severe brain injury in survivors (intraventricular hemorrhage grade ≥ 3 or periventricular leukomalacia). We performed random-effects meta-analyses, generating odds ratios with 95% confidence intervals for the first and second twin separately, and for both twins together. We assessed the risk of bias using a modified Newcastle Ottawa Scale (NOS) for observational studies and used Grading of Recommendations Assessment, Development and Evaluation approach (GRADE).

**Results:**

Our search generated 2695 articles, and after duplicate removal, we screened 2051 titles and abstracts, selecting 113 articles for full-text review. We contacted 36 authors, and ultimately, three observational studies met our inclusion criteria. In cephalic/non-cephalic twin pairs delivered by caesarean section compared to vaginal birth at 24^+0^–27^+6^ weeks the odds ratio for our composite outcome of neonatal death and severe brain injury for the cephalic first twin was 0.35 (95% CI 0.00–92.61, two studies, I^2^ = 76%), 1.69 for the non-cephalic second twin (95% CI 0.04–72.81, two studies, I^2^ = 55%) and 0.83 for both twins (95% CI 0.05–13.43, two studies, I^2^ = 56%). According to the modified Newcastle Ottawa Scale we assessed individual study quality as being at high risk of bias and according to GRADE the overall evidence for our primary outcomes was very low.

**Conclusion:**

Our systematic review on the safest mode of delivery for extremely preterm cephalic/non-cephalic twin pairs found very limited existing evidence, without significant differences in neonatal death and severe brain injury by mode of delivery.

**Electronic supplementary material:**

The online version of this article (10.1186/s12884-017-1554-7) contains supplementary material, which is available to authorized users.

## Background

Extreme prematurity, the birth of an infant before 28 weeks’ gestation, contributes significantly to infant mortality [1] and childhood morbidity [2]. Extremely preterm births represent approximately 0.2% of singleton births [3, 4], but 4.1% of twins births [5]. In extremely preterm twins from 24 to 27 weeks, the most frequent combination of presentations is cephalic/non-cephalic (42.5%) followed by cephalic/cephalic (25.3%) and non-cephalic/non-cephalic (22.6%) which differs significantly from term proportions [6].

Controversy remains as to the influence of mode of delivery on neonatal outcomes in extremely preterm singletons and twins in general. Some [7, 8], but not all studies [9] have raised concerns about the safety of vaginal birth for extremely preterm breech singleton infants. In a recent meta-analysis, Grabovac et al. 2017 found that caesarean delivery was associated with a 40% decrease in the odds of mortality and 40% decrease in odds of severe intraventricular hemorrhage (IVH; grades ≥3) in extremely preterm breech singletons who were actively resuscitated [10].

In twins, determining the safest mode of delivery is more complex than in singletons, since beyond gestational age, birth order and various presentation combinations need to be considered. For cephalic/non-cephalic twins above 32 weeks, the 2013 randomized controlled trial by Barrett et al. found that trial of labor is safe [11], but due to their inclusion criteria, could not provide guidance on twin birth before 32 weeks.

While a caesarean section is typically performed when the first twin presents as non-cephalic, vaginal birth is generally attempted when both twins are cephalic [12–15]. When the first twin is cephalic and the second is non-cephalic, there is less clinical consensus, leading to variations in clinical practice depending on the clinicians’ level of experience, training, and the prevailing obstetrical culture in their location of practice [12–15].

When the second twin is breech, the delivery involves a sequence of events which differs from a singleton breech delivery, including delivery through an already dilated cervix, potential for manoeuvres such as breech extraction and external cephalic version, precluding direct extrapolation of singleton data to the mode of delivery of twins. In face of all these considerations, we decided to perform a systematic review of the literature to assess the evidence regarding the safest mode of delivery of extremely preterm cephalic/non-cephalic twin pairs who were actively resuscitated.

## Methods

We planned to follow the Cochrane Handbook for Systematic Reviews of Interventions (Version 5.1.0) for randomized studies and the PRISMA guidelines for observational studies [16, 17]. We registered this protocol on PROSPERO (CRD42017056295).

### Information sources

We developed separate search strategies with the assistance of an experienced librarian for each database, consisting of medical subject headings (MeSH) and multipurpose terms (.mp), which we used to search Cochrane CENTRAL, MEDLINE and EMBASE from January 1,1994 - the year the guidelines for the use of antenatal corticosteroid were published -, until January 12, 2017, without language restriction (Additional file 1). We also searched for unpublished randomized controlled trials (RCT) on http://clinicaltrials.gov using the keywords “twin”, “twins”, “multiple pregnancy” and “multiple pregnancies”. We imported all references into a bibliographic software (Endnote X8). We manually searched the references of included studies and relevant systematic reviews for additional articles. We consulted a Maternal Fetal Medicine expert for knowledge on other studies published in this area.

### Eligibility

We planned to include all published randomized controlled trials and observational studies (cohort and case-control) comparing mode of delivery in extremely preterm (22^+0^ and 27^+6^ weeks) dichorionic or monochorionic-diamniotic twins presenting as a cephalic/non-cephalic pair who were actively resuscitated. If a study focused on mode of delivery in twins, but did not stratify the data according to gestational age or presentation, we contacted the authors to obtain these data. If the study population was defined by birth weight only, without data on gestational age, we included twins weighing ≤1000 g, which is approximately the 90th percentile for twins born at 27 weeks [18].

We excluded other types of publications (e.g. reviews, editorials, commentaries, case studies, conference proceedings, studies published only as abstracts, etc.). We excluded studies with insufficient data, such as <10 twin pairs total or less than five twin pairs per comparison group (i.e. caesarean and vaginal delivery). We excluded studies with data collected prior to 1994, regardless of publication date. If the data spanned 1994, we contacted the authors to confirm antenatal corticosteroid (ANCS) use was the standard of practice at the time of data collection, and included the study if either the authors confirmed that this was the case or were able to provide separate data for after 1994.

We focused on high-income countries, as they routinely provide active resuscitation for all infants ≥25 weeks, and variably offer resuscitation at 22, 23 or 24 weeks [19], while middle- and low-income countries typically do not. If data on active resuscitation were not provided in the study, we contacted the author to confirm that active resuscitation was planned for all included infants or to request separate data for the actively resuscitated infants only. If the author did not respond, but the study originated from a high-income country, we assumed that resuscitation was planned for all neonates ≥25 weeks. For middle and low incomes countries, if the author did not respond, we excluded the study.

We planned to exclude monochorionic-monoamniotic twins, conjoined twins, twins resulting from fetal reduction of a higher-order pregnancy, twin pairs with one or two fetal deaths before labour, twins with congenital anomalies and asynchronous delivery of the second twin where the aim was to prolong the pregnancy. We intended to exclude twins delivered by caesarean section as a result of an absolute contra-indication to vaginal delivery (e.g. fetal compromise before labour, fetal congenital anomaly, placenta or vasa praevia, uterine rupture, etc.).

Our primary outcome was a composite consisting of: 1) neonatal death defined as death in the first 28 days of life [20] and/or 2) severe brain injury (SBI) [21] among survivors, defined as severe intraventricular hemorrhage (IVH grades ≥3 based on Papile’s grading) or periventricular leukomalacia (PVL).

Our main secondary outcomes were the components of our primary composite outcome examined individually: neonatal death, and in survivors, severe brain injury. Another main secondary outcome was overall perinatal mortality (intrapartum death and neonatal death). We examined these outcomes separately in each twin individually according to birth order, and in both twins as pairs together. Our other infant and maternal secondary outcomes are presented in Additional file 2.

### Data collection

Two reviewers (CD and AMLM) independently reviewed the titles, abstracts, and full texts. As there are known issues with the kappa statistic (low kappa despite high agreement), we calculated percent agreement to assess inter-reviewer agreement on study inclusion. We used a piloted data collection form to extract data on baseline characteristics, exposures of interest, outcomes, and risk of bias assessment. Discrepancies between reviewers were resolved through discussion, with a third reviewer (SDM) available if necessary.

### Risk of bias assessment

We planned to use the Cochrane Collaboration’s Risk of Bias (RoB) tool for randomized control trials and the modified Newcastle-Ottawa Scale (NOS) for observational studies to assess risk of bias of our included studies [16, 22].

The Newcastle Ottawa Scale uses three categories, Selection, Comparability and Outcomes, to assess bias in observational studies. We modified the Selection and Outcomes categories by removing 1) ascertainment of exposure, since our exposures of interest (e.g. caesarean section or vaginal delivery) was only obtained through a secure medical record, 2) demonstration that the outcome of interest was not present at the beginning of the studies as the infant and maternal outcomes would not have been present at the time of the caesarean section or vaginal delivery, and 3) whether follow-up was long enough for the outcomes to occur because our outcomes of interest are assumed to have occurred after birth and before discharge from the hospital. We modified the Comparability category so that four points would be awarded for addressing key potential confounders. Those were identified in consultation with Maternal Fetal Medicine and Neonatology experts and were 1) caesarean section for fetal distress, 2) outborn status, 3) antenatal corticosteroid administration (ANCS) and 4) clinical chorioamnionitis. The study was awarded one point for each confounder it addressed for a maximum of four points. Hence, our modified scale awarded up to eight points in total. Since there are no validation studies on a modified scale, we determined that a study scoring eight points would be considered a high-quality study at low risk of bias. A study scoring seven points would be considered of moderate quality and at moderate risk of bias and a study scoring six points or less of low quality and at high risk of bias.

### Data analysis

Since we expected between-study heterogeneity, we performed random-effects meta-analyses, generating odds ratios (OR) and 95% confidence intervals (95% CI). We assessed heterogeneity using I^2^ statistic; we considered I^2^ values 0–40% to be low, 30–60% moderate, 50–90% substantial and 75–100% considerable heterogeneity [16, 17]. We intended to analyze our primary composite and main secondary outcomes according to pre-planned gestational week categories 22^+0^–23^+6^, 24^+0^–25^+6^ and 26^+0^–27^+6^ weeks and pooled together. We planned to separately pool RCT and observational data, to separately pool adjusted and unadjusted data, to separately analyze data from middle- and low-income countries, and to calculate the number needed to treat (NNT) for significant outcomes. All analyses were performed using Review Manager (RevMan) Version 5.3 [23].

### Risk of bias across studies

We used the Grading of Recommendations, Assessment, Development, and Evaluation (GRADE) system to assess the overall quality of evidence for our primary outcome, i.e. the confidence that an outcome’s effect size is close to the intervention’s true effect [22].

We used the GRADE system to rate the quality of evidence for each outcome as high, moderate, low or very low. GRADE recommends that RCTs start as high-quality evidence and observational studies as low-quality evidence, which is then either downgraded (RCTs and observational) or upgraded (observational). The evidence is downgraded by the presence of risk of bias, inconsistency, indirectness, imprecision or publication bias. We assessed those in the following manner: 1) for RCTs, we planned to assess risk of bias using the Cochrane’s RoB and for observational studies using the modified NOS; 2) Inconsistency was assessed by substantial heterogeneity as indicated by I^2^ values above 50%; 3) Indirectness was assessed by differences in the population, intervention, or outcome, or indirect comparison; 4) Imprecision was assessed by checking whether 95% CIs overlap no effect and/or fail to exclude important benefit/harm; 5) Assessment of publication bias was planned with funnel plots for outcomes with 10 studies or more [24]. The evidence is upgraded in the presence of a large effect, dose-response effect and if all potential confounding would minimize the demonstrated effect.

### Subgroup analyses

In addition to stratification by gestational age categories, we intended to separately analyze our main secondary outcomes by birth weight categories (<500 g, 500–999 g and <1000 g). We intended to include the infants who died intrapartum in the adjusted analysis if active resuscitation was planned for them, as excluding them could result in overestimation of the benefits of either mode of delivery.

Data permitting, we intended to address the a priori selected key confounders previously mentioned through subgroup analysis: caesarean section for fetal distress, outborn status, ANCS administration and clinical chorioamnionitis. We also intended to collect information on other potential confounders: cause of prematurity, gestational age at preterm premature rupture of the membranes (PPROM), presence of abruption, presence of growth restriction, weight discrepancy between twins, length of labour, length of birth interval between twins, magnesium sulfate administration before birth, surfactant administration after birth, exact presentation of second twin (breech vs transverse), and maneuvers required for vaginal delivery of the second twin (e.g. external cephalic version versus breech extraction).

We intended to perform a sensitivity analysis by removing low and moderate quality studies to obtain effect estimates using high quality studies only. We also planned to perform a sensitivity analyses by removing studies that excluded intrapartum fetal demise from their study population.

## Results

### Search strategy

Our search retrieved a total of 2695 abstracts (Cochrane CENTRAL = 193, MEDLINE = 611, EMBASE = 1881, Fig. 1). We identified an additional prospective cohort study from http://clinicaltrials.gov, with a planned subgroup analysis for twins less than 28 weeks, however the initial publication included solely data for twins more than 32 weeks [25], and the authors were unable to provide additional data after contact.

**Fig. 1:**
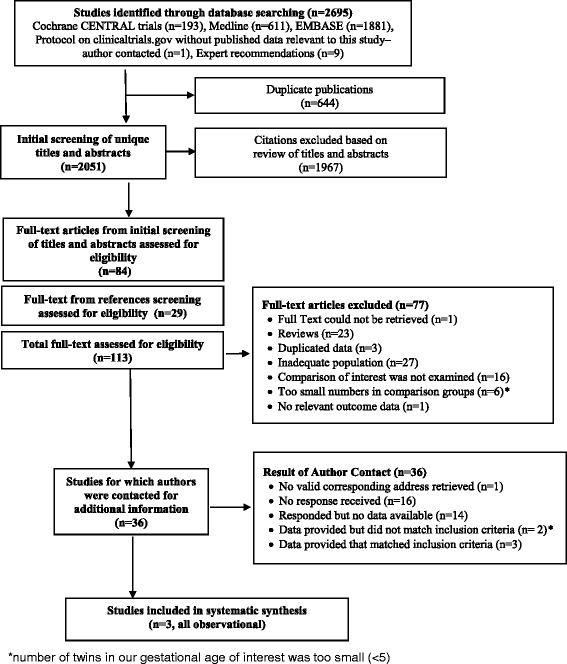
Study flowchart for a systematic review/meta-analyses on the safest mode of delivery for extremely preterm cephalic/non-cephalic twin pairs

After removing duplicates, we screened 2051 titles and abstracts, selecting 113 full-text articles for review. The initial agreement between reviewers was 92% for full text review. We preliminarily included 36 studies, whose authors we contacted to obtain additional information (Fig. 1, Additional file 3). The response rate was 54%. Out of the 19 authors who responded, 14 could not provide the requested data [26–39], two authors contributed data we could not use [40, 41], and three authors provided data we included in our meta-analyses [42–44]. The main reasons for not being able to include studies without author response were absence of stratification of data by gestational age < 28 weeks or birth weight < 1000 g, by birth order or by presentation in the original paper (Additional file 3). For the 16 out of 19 authors (84%) who responded favorably to our request for additional information, the reason for not being able to provide data or include the provided data in the study were the same (Additional file 3).

### Description of studies

We included three observational studies from high-income countries: France, Israel and Slovenia. The analyzed data were collected between 2003 and 2012 (Table 1). The comparison in all three studies was mode of delivery - caesarean section or vaginal delivery - for both twins.

**Table 1:** Study characteristics in a systematic review/meta-analyses on the safest mode of delivery for extremely preterm cephalic/non-cephalic twin pairs

Author, Publication Year, Country; Study Period; Study Design	Inclusion and exclusion criteria	Usual practice regarding twin delivery	Outcomes in original study^¥^
**Boukerrou, 2011** France; 2006–2011; Prospective cohort	**Inclusion:** All twin births during the study period **Exclusion:** HOM, stillbirths, births less 24 weeks	For non-cephalic second twin, breech extraction with or without internal podalic version is preferred.	**Neonatal death (0–28 days), graded IVH and PVL** (in provided data only)
**Barzilay, 2015** Israel; 2004–2011; Retrospective cohort	**Inclusion:** All twin births with second twin birthweight less 1500 g **Exclusion:** Birth less 24 weeks, fetal death of one or both twins, major malformation in one or both twins	Allow vaginal delivery of cephalic-non-cephalic twin pairs regardless of EFW or GA if EFW of twin B is not 20% higher than that of twin A. Breech extraction is preferred for delivering non-cephalic twin B.	Apgar 5 min, Cord blood PH, **Neonatal death (not otherwise specified),** Birth trauma, **RDS,** Sepsis, NEC, **IVH, Composite adverse neonatal outcome** (neonatal death, RDS, sepsis, NEC, or IVH grade ≥ 3)
**Bricelj, 2016** Slovenia; 2003–2012; Retrospective cohort	**Inclusion:** All deliveries from 22 weeks or birth weight 500 g up to less than 37 weeks **Exclusion:** Delayed births, combined deliveries, stillbirths (in provided data only)	Not stated	TTN, **RDS, Ventilation need**

*HOM* high order multiple pregnancies, *EFW* estimated fetal weight, *GA* gestational age, *IVH* intraventricular hemorrhage, *PVL* periventricular leukomalacia, *RDS* respiratory distress syndrome, *NEC* necrotizing enterocolitis, *TTN* transient tachypnea of the newborn ^¥^ Outcomes provided by the authors for twins less 28 weeks are **bolded**

Outcomes were not stratified for our population of interest in the original studies, namely cephalic/non-cephalic twin pairs <28 weeks, and hence the data were provided to us by all three authors upon request. Gestational age ranged from 22^+0^–27^+6^ weeks across the three studies, and the outcomes were provided separately for each twin. One set of twins underwent a combined delivery, whereby the first twin was delivered vaginally and the second twin by caesarean section in Boukerrou 2011 [43]. The outcomes for this twin pair were included in the respective mode of delivery of each of the twin. We initially planned to include studies with at least 10 twin pairs, however, due to the paucity of studies meeting our inclusion criteria, and in order to maximize the number of twins, we included studies with eight or more twin pairs.

### Risk of bias assessment

According to our modified NOS, all three studies were at high risk of bias, scoring three points out of the maximum of eight (Table 2). All studies lost one point in the Outcome category, as they did not account for loss to follow-up. None of the studies addressed any of the key confounders for our outcome of interest (i.e. caesarean section for fetal distress, outborn status, ANCS use and clinical chorioamnionitis), and hence no points were allotted for the Comparability criteria.

**Table 2:** Bias assessment in a systematic review/meta-analyses on the safest mode of delivery for extremely preterm cephalic/non-cephalic twin pairs

Study ID Author, Year, Country	Total	Selection		Outcome		Comparability			
		Representativeness of the exposed cohort	Selection of the non- exposed cohort	Assessment of outcome	Adequacy of follow up of cohorts	Emergent caesarean for fetal distress	Clinical Chorioamnionitis	Outborn Status	ANCS
Boukerrou, 2011 France	3/8	★	★	★	**–**	**–**	**–**	**–**	**–**
Barzilay, 2015 Israel	3/8	★	★	★	**–**	**–**	**–**	**–**	**–**
Bricelj, 2016 Slovenia	3/8	★	★	★	**–**	**–**	**–**	**–**	**–**

*ANCS* antenatal corticosteroids, ★ = 1 point awarded, “- “= no points awarded. Assessed risk of bias of observational studies using a modified Newcastle Ottawa Scale

All twins from Boukerrou [43] and Barzilay [44] were planned for active resuscitation. Bricelj 2016 [42] could not confirm active resuscitation for all twins, and therefore we included their data only for twins born at 25^+0^–27^+6^ weeks in our analysis.

### Effects of mode of delivery

**Fig. 2:**
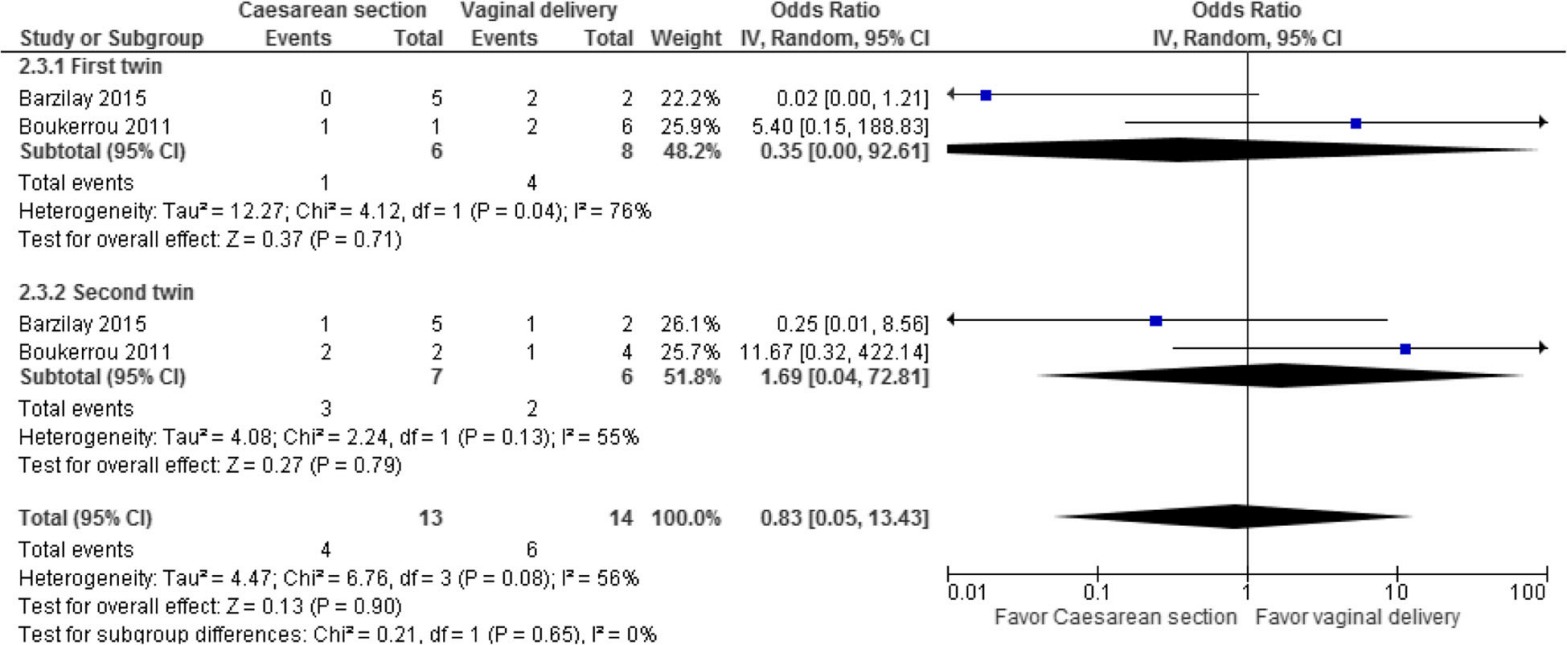
Composite outcome in a systematic review/meta-analyses on the safest mode of delivery for extremely preterm cephalic/non-cephalic twin pairs. SBI – Severe brain injury defined as intraventricular hemorrhage grade ≥3 or periventricular leukomalacia; IV – inverse variance; CI – confidence interval; I^2^-heterogeneity. Composite outcome consists of neonatal death or severe brain injury (SBI) in survivors, at 24^0^–27^6^ weeks’ gestation by mode of delivery

In cephalic/non-cephalic twin pairs delivered by caesarean section compared to vaginal birth at 24^+0^–27^+6^ weeks the odds ratio for our composite outcome of neonatal death and severe brain injury for the cephalic first twin was 0.35 (95% CI 0.00–92.61, two studies, I^2^ = 76%, Fig. 2, Table 3), 1.69 for the non-cephalic second twin (95% CI 0.04–72.81, two studies, I^2^ = 55%) and 0.83 for both twins (95% CI 0.05–13.43, two studies, I^2^ = 56%).

**Table 3:** Outcomes in a systematic review/meta-analyses on the safest mode of delivery for extremely preterm cephalic/non-cephalic twin pairs

Outcome	GA category (weeks)	Number of studies		CS (n/N)	VD (n/N)	OR (95% CI) for CS	I^2^ (%)	GRADE Quality of the evidence*
Neonatal death or Severe Brain Injury in survivors	24^+0^–27^+6^	2	*First twin* (cephalic)	1/6	4/8	OR 0.35 (0.00–92.61)	76	Very Low
			*Second twin* (non-cephalic)	3/7	2/6	OR 1.69 (0.04–72.81)	55	
			*Both twins*	4/13	6/14	OR 0.83 (0.05–13.43)	56	
Neonatal death	24^+0^–27^+6^	2	*First twin* (cephalic)	0/7	2/10	OR 0.36 (0.03–4.40)	0	Very low
			*Second twin* (non-cephalic)	2/8	2/9	OR 1.31 (0.02–79.60)	66	
			*Both twins*	2/15	4/19	OR 0.73 (0.10–5.46)	26	
Severe Brain Injury in survivors	24^+0^–27^+6^	2	*First twin* (cephalic)	1/6	2/6	OR 0.59 (0.00–154.35)	74	Very low
			*Second twin* (non-cephalic)	1/5	0/4	OR 1.00 (0.02–40.28)	N/A	
			*Both twins*	2/11	2/10	OR 0.76 (0.03–17.34)	48	
Respiratory distress syndrome (RDS)	25^+0^–27^+6^	2	*First twin* (cephalic)	13/14	15/15	OR 0.23 (0.01–6.25)	N/A	Very low
			*Second twin* (non-cephalic)	13/14	13/15	OR 1.60 (0.12–20.99)	N/A	
			*Both twins*	26/28	28/30	OR 0.77 (0.10–5.87)	0	

*GA* gestational age, *CS* caesarean section, *VD* vaginal delivery, *n* number of cases within exposure group, *N* total number in exposure group, *OR* odds ratio, *CI* confidence interval, Severe Brain Injury defined as intraventricular hemorrhage grade ≥ 3 or periventricular leukomalacia, *N/A* not applicable. *Based on the Grading of Recommendations Assessment, Development and Evaluation quality of evidence assessment (GRADE) approach

**Fig. 3:**
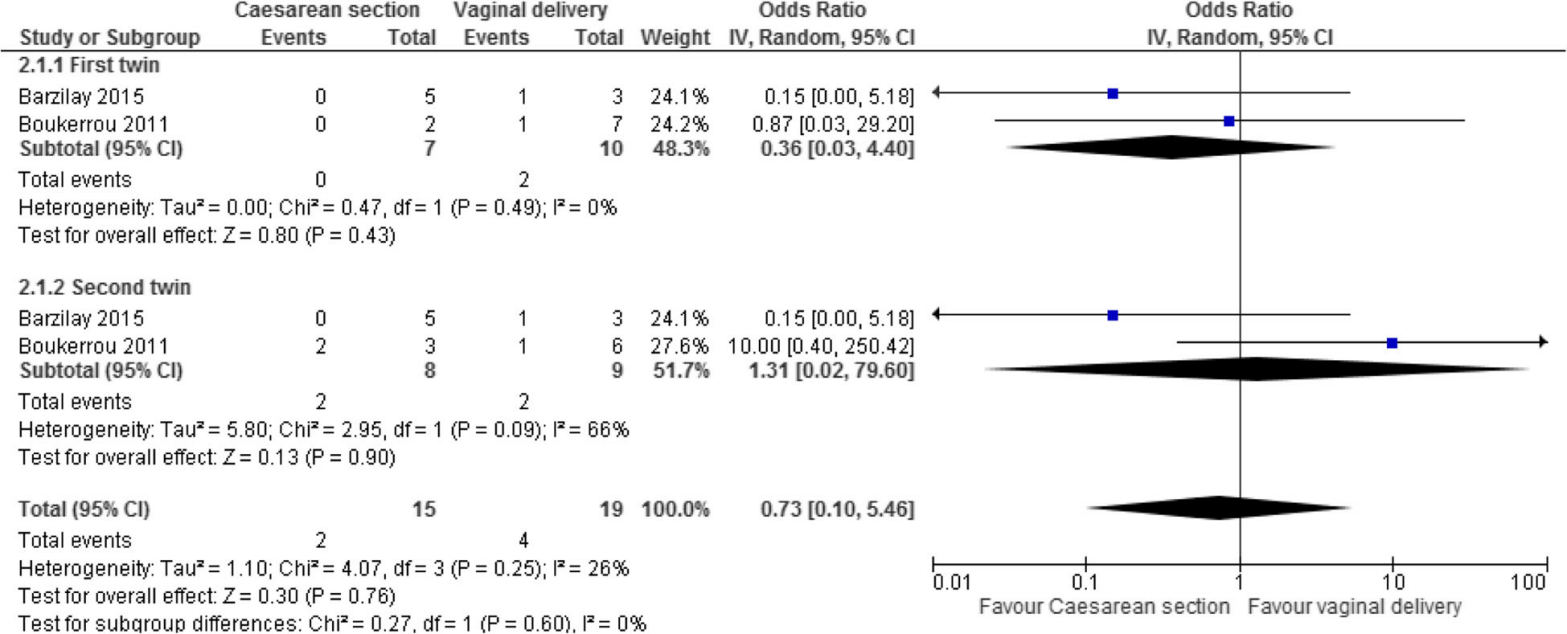
Neonatal death in a systematic review/meta-analyses on the safest mode of delivery for extremely preterm cephalic/non-cephalic twin pairs. IV – inverse variance; CI – confidence interval; I^2^-heterogeneity. Neonatal death at 24^0^–27^6^ weeks’ gestation by mode of delivery

The odds ratios of neonatal death were for twins delivered between 24^+0^–27^+6^ weeks by caesarean section compared to vaginally were 0.36 for the cephalic first twins (95% CI 0.03–4.40, two studies, I^2^ = 0%, Fig. 3), 1.31 for the non-cephalic second twins (95% CI 0.02–79.60, two studies, I^2^ = 66%), and 0.73 for both twins together (95% CI 0.10–5.46, two studies, I^2^ = 26%).

**Fig. 4:**
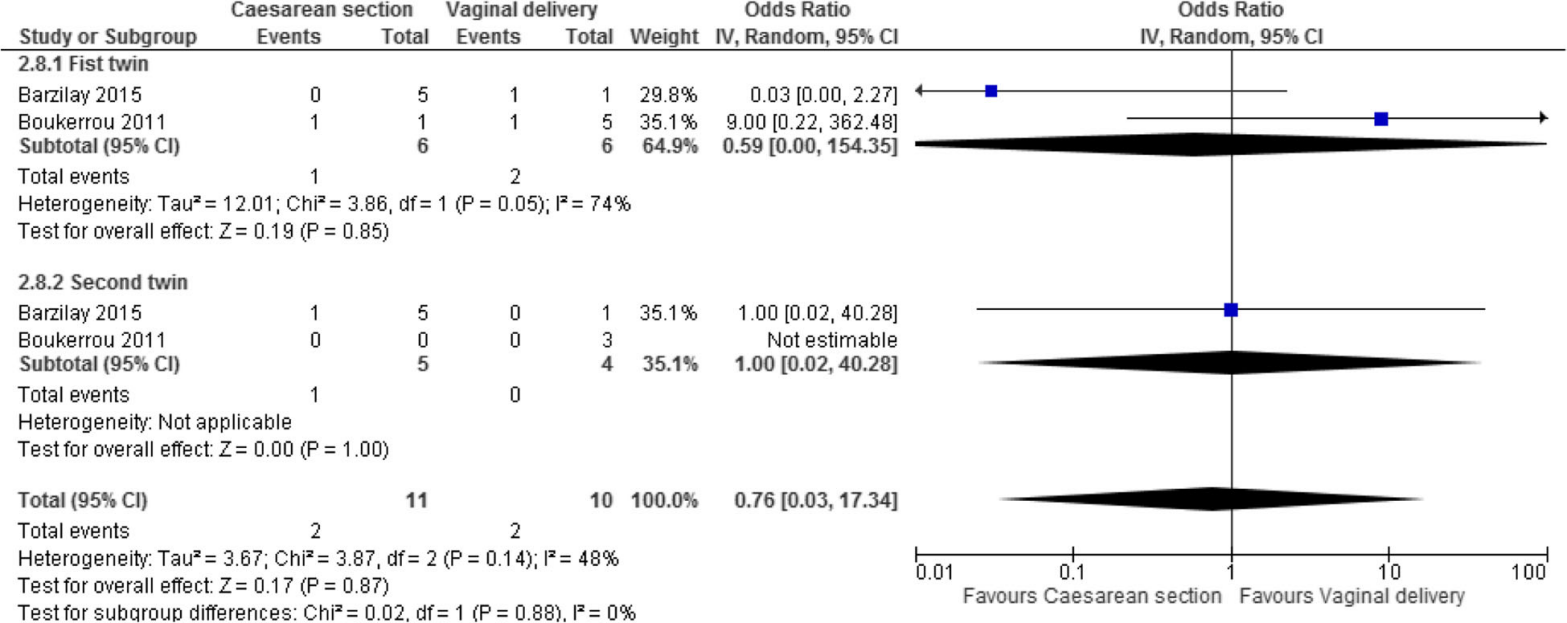
Severe brain injury in a systematic review/meta-analyses on the safest mode of delivery for extremely preterm cephalic/non-cephalic twin pairs. IV – inverse variance; CI – confidence interval; I^2^ - heterogeneity. Severe brain injury at 24^0^–27^6^ weeks’ gestation by mode of delivery

The odds ratios of severe brain injury in survivors, for twins delivered between 24^+0^–27^+6^ weeks by caesarean section compared to vaginally were 0.59 for the cephalic first twins (95% CI 0.00–154.35, two studies, I^2^ = 74%, Fig. 4), 1.00 for the non-cephalic second twins (95% CI 0.02–40.28, two studies, I^2^ = N/A), and 0.76 for both twins together (95% CI 0.03–17.34, two studies, I^2^ = 48%). Data on severe brain injury were missing for five surviving twins out of fourteen (36%) in the data by Boukerrou 2011 [43] and for two surviving twins out of fourteen in the data from Barzilay 2015 [44].

**Fig. 5:**
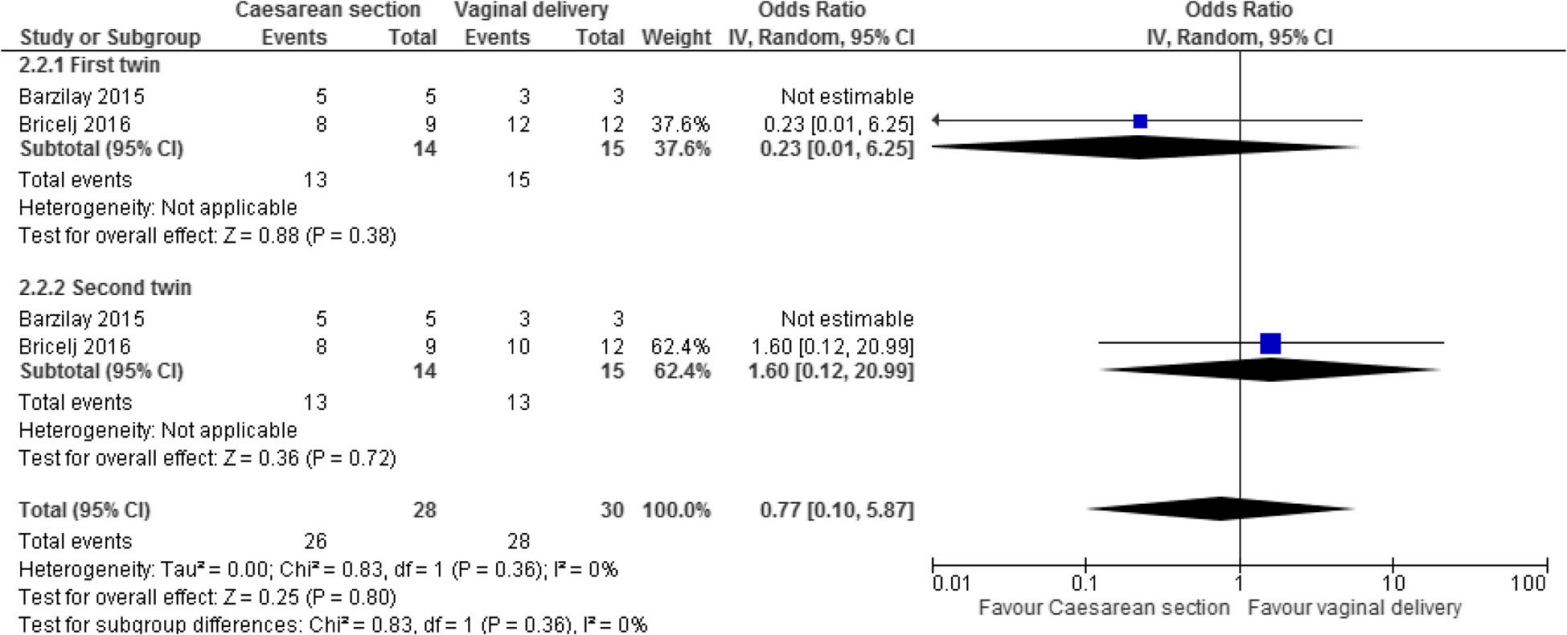
Respiratory distress syndrome in a systematic review/meta-analyses on the safest mode of delivery for extremely preterm cephalic/non-cephalic twin pairs. IV – inverse variance; CI – confidence interval; I^2^ - heterogeneity. Respiratory distress syndrome at 25^0^–27^6^ weeks’ gestation by mode of delivery

For our secondary outcomes, the odds ratios of respiratory distress syndrome for twins delivered between 25^+0^–27^+6^ weeks by caesarean section compared to vaginally were 0.23 for the cephalic first twins (95% CI 0.01–6.25, two studies, I^2^ = N/A, Fig. 5), 1.60 for the non-cephalic second twins (95% CI 0.12–20.99, two studies, I^2^ = N/A), and 0.77 for both twins together (95% CI 0.10–5.87, two studies, I^2^ = 0%). Data for other secondary infant and maternal outcomes were not available.

We were unable to stratify any of our outcomes by gestational age categories, as the sample sizes were too small. We could not perform any of the planned subgroup analyses due to lack of data in primary studies. Since all studies were at high risk of bias, we could not perform the planned sensitivity analyses. We were unable to pool adjusted data, since that data was lacking for our population of interest in the primary studies.

### Quality of the evidence (GRADE)

We downgraded the quality of evidence due to serious risk of bias and imprecision, but not due to inconsistency and indirectness. Publication bias could not be assessed, as the number of studies was <10 per outcome. We could not upgrade the evidence, as a large effect was not present, and confounding was not accounted for; the dose-response was not applicable for our meta-analyses. The overall quality of evidence was very low for our primary composite outcome (neonatal death or SBI), for neonatal death and for respiratory distress syndrome (Table 3, Additional file 4).

## Discussion

### Main findings

In this systematic review on the safest mode of delivery for extremely preterm cephalic/non-cephalic twin pairs, we found scarce data. Analysis did not favour either caesarean section or vaginal birth. The confidence intervals were wide and encompassed one for our primary composite outcome of neonatal death and severe brain injury, as well as for neonatal death alone or severe brain injury alone for the cephalic first twins, non-cephalic second twins and when both twins were considered together.

### Strengths and limitations

This systematic review has some strengths, including its focus on a specific clinical dilemma for which consensus is lacking thus far. We strove to provide a clinically relevant assessment of the evidence regarding the safest mode of delivery for extremely preterm twins, and therefore we accounted for gestational age, birth order and presentation of each twin in our study design, as each of those could impact neonatal outcomes. We aimed at controlling the four major confounders of outcome in extremely preterm births (caesarean section for fetal distress, outborn status, ANCS use and clinical chorioamnionitis). Furthermore, the safest mode of delivery for twins cannot be inferred from singleton data as twinning itself may affect outcomes [45] and outcomes in both twins have to be looked at since the impact on both should be considered when choosing a mode of delivery.

Our systematic review also has limitations, the main one being the lack of primary randomized data on the safest mode of delivery of extremely preterm infants. Although the most ideal study design would be a randomized controlled trial, previous RCTs in singletons have failed [46–48], and hence it is unlikely that another large-enough RCT will be mounted in the near future. We therefore must rely on alternative research methodology. The observational data were scarce in terms of the number of extremely preterm twins available for analyses, which may be the reason for lack of significant differences, even in our pooled data, between outcomes after caesarean and vaginal delivery. Moreover, although we addressed the most important confounder, active resuscitation, by requiring it for inclusion in the analysis, other key confounders were not available for our subpopulation in the primary studies, which may also contribute to the lack of significant differences. Additionally, over the 1994–2017 period, the quality and accuracy of ultrasound estimation of gestational age varied. It would have been preferable to have data according to planned mode of delivery, rather than actual, but this was not available.

Lastly, maybe the most striking limitation was the overall sparsity of available data from primary studies that met our inclusion criteria.

### Comparing our findings to existing literature

In comparing our results to previous reviews on the impact of mode of delivery in twins, we found that some previous systematic reviews did not focus on the extreme preterm period, but rather on twins above 32 weeks of gestation or above 1500 g [49, 50], while others were unable to control for gestational age in their analysis [51]. To our knowledge, only narrative reviews have addressed the mode of delivery of extremely preterm twins. The first one included data from the 1980’s and early 1990’s, which are not as relevant to current clinical decision making given the subsequent advances in neonatal management and survival [52]. Nevertheless, these authors concluded that: “*Management of low birth weight nonvertex second twins remains controversial… The retrospective nature and possibility for type II error of the majority of studies examining safety of vaginal delivery of the LBW nonvertex second twin makes definitive conclusions regarding vaginal delivery of these infants difficult*.” A more recent review encompassed three additional studies from the 2000’s, but none of those stratified outcomes for twins below 1500 g or 34 weeks [53]. The authors concluded again that: “*The optimal mode of delivery in the preterm twin gestation (particularly those less than 2000 g) continues to be debated, data… remains limited*.”

Some individual cohort studies addressing the safest mode of delivery for first or second twins, with a birth weight below 1500 g or a gestational age less than 34 weeks have found a decrease in risk of death and/or morbidity with caesarean section [54–59] while others have not [28–30, 44, 60–65]. However, these studies neither stratified by gestational age less than 28 weeks nor by extremely low birth weight (< 1000 g), nor by presentation of the second twin.

In extremely preterm twins, prior to 1994 and hence advent of antenatal corticosteroid therapy and other advances in neonatal management, some studies had found a decrease in the risk of mortality with caesarean section in second twins weighing 601–999 g [66] and those weighing less than 1000 g [67]. More recently, Thomas 2016 [68] found an increase in survival with caesarean section for multiples from 24 to 26 weeks’ gestation presenting as non-footling breech, but that difference was no longer significant after adjustment for gestational age, chorioamnionitis and maternal age. This study did not stratify data by birth order, and included higher order multiples.

Garite 2004 [69] also found no significant difference in mortality according to mode of delivery in 24–26 week twins stratified by birth order but not by presentation. They hypothesized: “*It may also be that adverse outcomes, which tend to dominate most studies, in second twins that are seen in vaginal deliveries may be related primarily to term or near-term babies.*” This would go along with our analyses, even though not significant, in which the point estimate favoured caesarean section for the cephalic first twins as well as when all twins were considered together but not for the second twin.

Yang 2005 [54] focused on non-cephalic second twins, and stratified for those weighing less than 500–1499 g, concluding that vaginal delivery increased the risk of mortality compared to caesarean section, even when comparing to caesarean section performed for the second twin in the context of a combined delivery. Wen 2004 [56], studying second twins in any presentation, found the same protective effect of caesarean section below 36 weeks, but not after 36 weeks, when the only increase in mortality for the second twin was in fact in the case of combined delivery. These observations suggest that in very preterm twins and likely extremely preterm twins, in contrast to higher gestational ages, the mode of delivery may interact in a different manner with birth order and presentation to influence mortality and morbidity. The exact gestational age at which such an interaction tips is unknown and warrants more research.

## Conclusion

In this systematic review of the safest mode of delivery for extremely preterm cephalic/non-cephalic twin pairs, we did not find a significant reduction in the odds of our composite outcome, neonatal death and severe brain injury, with either mode of delivery. The extremely limited primary and clinically relevant data available highlights the need for further appropriately designed research regarding safest mode of delivery for extremely preterm twins. An appropriate method would have to include details relevant to clinical decision making in that field.

Future research should seek to understand the long term neurodevelopmental outcomes and maternal outcomes in relation to mode of delivery of extremely preterm twins.

## Additional files

Additional file 1: Search strategy for a systematic review and meta-analysis on the safest mode of delivery for extremely preterm cephalic/non-cephalic twin pairs. (DOC 124 kb)

Additional file 2: Secondary infant and maternal outcomes included in a systematic review and meta-analyses on the safest mode of delivery for extremely preterm cephalic/non-cephalic twin pairs. (DOC 31 kb)

Additional file 3: Summary table of excluded studies in a systematic review and meta-analyses on the safest mode of delivery for extremely preterm cephalic/non-cephalic twin pairs - author contacted but could not provide the necessary data or did not respond. (DOC 179 kb)

Additional file 4: GRADE assessment for the primary composite outcome (neonatal death and severe brain injury), neonatal death and respiratory distress syndrome in a systematic review and meta-analyses for the safest mode of delivery for extremely preterm cephalic/non-cephalic twin pairs. (DOC 61 kb)
