# Supplementary material for: What is the safest mode of delivery for extremely preterm cephalic/non-cephalic twin pairs? A systematic review and meta-analyses
Source: BMC Pregnancy Childbirth. 2017 Nov 29;17:397. doi: 10.1186/s12884-017-1554-7 (PMC5707900; doi:10.1186/s12884-017-1554-7)
Supplement: Supplementary file 1 — Search strategy for a systematic review and meta-analysis on the safest mode of delivery for extremely preterm cephalic/non-cephalic twin pairs. (DOC 124 kb) [file 12884_2017_1554_MOESM1_ESM.doc]

# **Appendix 1** – Search strategy for a systematic review and meta-analysis on the safest mode of delivery for extremely preterm cephalic/non-cephalic twin pairs

| **Database: OVID Medline Epub Ahead of Print, In-Process & Other Non-Indexed Citations, Ovid MEDLINE(R) Daily and Ovid MEDLINE(R)** <1946 to Jan/12 2017> | | |
| --- | --- | --- |
| **Numbers** | **Searches** | **Results** |
| 1 | multiple birth offspring/ or twins/ or twins, dizygotic/ or twins, monozygotic/ | 27984 |
| 2 | pregnancy, multiple/ or pregnancy, twin/ or superfetation/ | 11061 |
| 3 | twin:.mp. | 67204 |
| 4 | gemell:.mp. | 1335 |
| 5 | ((di adj 1 chorion:) or (bi adj 1 chorion:) or di-chorion: or bi-chorion: or dichorion: or bichorion:).mp. | 1084 |
| 6 | (monochorion: or mono-chorion: or (mono adj1 chorion:) or unichorion: or uni-chorion: or (uni adj1 chorion:)).mp. | 2190 |
| 7 | ((di adj1 amnio:) or (bi adj1 amnio:) or di-amnio: or bi-amnio: or diamnio: or biamnio:).mp. | 899 |
| 8 | (multi: adj2 (pregnan: or gestation: or birth:)).mp. | 18388 |
| 9 | (gemin: and pregnancy).mp. | 95 |
| 10 | or/1-9 | 79508 |
| 11 | parturition/ | 7978 |
| 12 | delivery, obstetric/ or vaginal birth after caesarean/ | 28670 |
| 13 | caesarean section/ or caesarean section, repeat/ | 42488 |
| 14 | extraction, obstetrical/ | 2525 |
| 15 | ((mode or route or way or type) adj2 (deliver: or birth: or parturition:)).mp. | 12407 |
| 16 | (cesar: or ceasar: or caesar: or c-section: or csection: or (c adj section:)).mp. | 69719 |
| 17 | (vaginal birth: or vaginal deliver:).mp. | 15111 |
| 18 | surgical birth:.mp. | 39 |
| 19 | (abdominal adj1 (birth: or deliver:)).mp. | 542 |
| 20 | ((fet: adj extraction) or (foet: adj extraction) or breech extraction).mp. | 243 |
| 21 | or/11-20 | 108491 |
| 22 | infant, very low birth weight/ or infant, extremely low birth weight/ or infant, extremely premature/ | 10637 |
| 23 | ((very adj1 preterm:) or (very adj1 prem:) or (extrem: adj1 preterm:) or (extrem: adj1 prem:)).mp. | 7640 |
| 24 | (periviab: or (peri: adj2 viab:) or peri-viab:).mp. | 766 |
| 25 | (vlbw: or elbw:).mp. | 4691 |
| 26 | (very adj1 low adj1 (birthweight or birth weight)).mp. | 12526 |
| 27 | (extremely adj1 low adj1 (birthweight or birth weight)).mp. | 3447 |
| 28 | ((22: adj1 week:) or (23: adj1 week:) or (24: adj1 week:) or (25: adj1 week:) or (26: adj1 week:) or (27: adj1 week:) or (28: adj1 week:)).mp. | 69252 |
| 29 | or/22-28 | 87665 |
| 30 | 10 and 21 and 29 | 718 |
| **31** | **limit 30 to yr="1994 -Current"** | **611** |

| **Database: EMBASE** <1974 to Jan/12 2017> | | |
| --- | --- | --- |
| **Numbers** | **Searches** | **Results** |
| 1 | twin:.mp. | 70778 |
| 2 | gemell:.mp. | 1615 |
| 3 | (dichorion: or bichorion: or (di adj1 chorion:) or (bi adj1 chorion:) or di-chorion: or bi-chorion:).mp. | 1578 |
| 4 | (di-amnio: or bi-amnio: or (di adj amnio:) or (bi adj amnio:) or diamnio: or biamnio:).mp. | 1307 |
| 5 | (monochorion: or mono-chorion: or (mono adj1 chorion:)).mp. | 2932 |
| 6 | (multi: adj2 (pregnan: or gestation: or birth:)).mp. | 22324 |
| 7 | (gemin: and pregnancy).mp. | 114 |
| 8 | twins/ or dizygotic twins/ or monozygotic twins/ | 43677 |
| 9 | twin pregnancy/ or multiple pregnancy/ | 22910 |
| 10 | or/1-9 | 87482 |
| 11 | ((mode or route or way or type) adj2 (deliver: or birth: or parturition:)).mp. | 16659 |
| 12 | (cesar: or ceasar: or caesar: or csection: or csection: or (c adj section:)).mp. | 95055 |
| 13 | (vaginal birth: or vaginal deliver:).mp. | 30211 |
| 14 | surgical birth:.mp. | 40 |
| 15 | (abdominal adj1 (birth: or deliver:)).mp. | 608 |
| 16 | (fet: extraction or foet: extraction or breech extraction).mp. | 1804 |
| 17 | birth/ | 15870 |
| 18 | obstetric delivery/ or vaginal birth after caesarean/ or vaginal delivery/ | 27161 |
| 19 | caesarean section/ or repeat caesarean section/ | 79889 |
| 20 | breech extraction/ | 1607 |
| 21 | or/11-20 | 133355 |
| 22 | ((very adj1 preterm:) or (very adj1 prem:) or (extrem: adj1 preterm:) or (extrem: adj1 prem:)).mp. | 8903 |
| 23 | (periviab: or (peri: adj2 viab:) or peri-viab:).mp. | 873 |
| 24 | (vlbw: or elbw:).mp. | 5802 |
| 25 | (very adj1 low adj1 (birthweight or birth weight)).mp. | 13535 |
| 26 | (extremely adj1 low adj1 (birthweight or birth weight)).mp. | 4051 |
| 27 | ((22: adj1 week:) or (23: adj1 week:) or (24: adj1 week:) or (25: adj1 week:) or (26: adj1 week:) or (27: adj1 week:) or (28: adj1 week:)).mp. | 89537 |
| 28 | prematurity/ | 96580 |
| 29 | very low birth weight/ or extremely low birth weight/ | 13524 |
| 30 | or/22-29 | 189122 |
| 31 | 10 and 21 and 30 | 2025 |
| **32** | **limit 31 to yr="1994 -Current"** | **1881** |

| **Database: Cochrane CENTRAL** <Jan/12 2017> | | |
| --- | --- | --- |
| **Numbers** | **Searches** | **Results** |
| 1 | MeSH descriptor: [Twins] explode all trees | 197 |
| 2 | MeSH descriptor: [Pregnancy, Twin] explode all trees | 36 |
| 3 | Twin* | 1795 |
| 4 | Gemell* | 271 |
| 5 | Multiple near/4 pregnanc* | 1183 |
| 6 | Multiple near/4 birth* | 419 |
| 7 | Multiple near/4 gestation* | 391 |
| 8 | di* near/4 chorion* | 153 |
| 9 | mono* near/4 chorion* | 3 |
| 10 | {or #1-#9} | 3234 |
| 11 | MeSH descriptor: [Delivery, Obstetric] explode all trees | 4826 |
| 12 | MeSH descriptor: [Caesarean Section] explode all trees | 2822 |
| 13 | cesar* | 7834 |
| 14 | caesar* | 3702 |
| 15 | c-section | 130 |
| 16 | abdominal near/4 deliver* | 98 |
| 17 | abdominal near/4 birth* | 26 |
| 18 | surgical near/4 birth* | 41 |
| 19 | surgical near/4 deliver* | 147 |
| 20 | deliver* obstetric* | 8653 |
| 21 | vagina* obstetric | 1612 |
| 22 | MeSH descriptor: [Labor Presentation] explode all trees | 174 |
| 23 | breech* | 360 |
| 24 | non near/4 vertex | 40 |
| 25 | non near/4 cephalic | 50 |
| 26 | extract* near/4 breech* | 26 |
| 27 | extract* near/4 fet* | 60 |
| 28 | {or #11-#27} | 15488 |
| 29 | MeSH descriptor: [Infant, Premature] explode all trees | 3303 |
| 30 | MeSH descriptor: [Infant, Extremely Premature] explode all trees | 92 |
| 31 | MeSH descriptor: [Infant, Extremely Low Birth Weight] explode all trees | 101 |
| 32 | MeSH descriptor: [Infant, Very Low Birth Weight] explode all trees | 870 |
| 33 | MeSH descriptor: [Obstetric Labor, Premature] explode all trees | 1289 |
| 34 | preterm* | 9035 |
| 35 | pre* near/4 term* | 13908 |
| 36 | prematur* | 17270 |
| 37 | pre* near/4 matur* | 564 |
| 38 | immatur* | 1134 |
| 39 | extrem* near/4 pre* | 1374 |
| 40 | 22* near/4 week* | 53077 |
| 41 | 23* near/4 week* | 2152 |
| 42 | 24* near/4 week* | 17854 |
| 43 | 25* near/4 week* | 4640 |
| 44 | 26* near/4 week* | 5706 |
| 45 | 27* near/4 week* | 2140 |
| 46 | 28* near/4 week* | 4627 |
| 47 | peri* near/4 viab* | 36 |
| 48 | periviab* | 5 |
| 49 | vlbw* | 900 |
| 50 | elbw* | 219 |
| 51 | {or #29-#50} | 99242 |
| 52 | **{and #10, #28, #51} Publication Year from 1994 to 2017, in Trials** | **193** |

| **Database: ClinicalTrials.gov**<Jan/12 2017> | | |
| --- | --- | --- |
|  | **Keywords** | **Results** |
| 1 | “Twin” or “Twins” | 1 |
| 2 | “Multiple pregnancy” or “Multiple pregnancies” | 0 |
| 3 | **Total** | **1** |
