# Supplementary material for: What is the safest mode of delivery for extremely preterm cephalic/non-cephalic twin pairs? A systematic review and meta-analyses
Source: BMC Pregnancy Childbirth. 2017 Nov 29;17:397. doi: 10.1186/s12884-017-1554-7 (PMC5707900; doi:10.1186/s12884-017-1554-7)
Supplement: Supplementary file 2 — Secondary infant and maternal outcomes included in a systematic review and meta-analyses on the safest mode of delivery for extremely preterm cephalic/non-cephalic twin pairs. (DOC 31 kb) [file 12884_2017_1554_MOESM2_ESM.doc]

# **Appendix 2** – Secondary infant and maternal outcomes included in a systematic review and meta-analyses on the safest mode of delivery for extremely preterm cephalic/non-cephalic twin pairs.

| **Secondary infant outcomes** | - Signs of intrapartum asphyxia (Apgar <4 at 5 minutes, umbilical arterial pH <7.0 or any author-defined asphyxia) - Bronchopulmonary dysplasia (BPD) or chronic lung disease defined as oxygen use (and/or positive pressure support) at 36 weeks postmenstrual age - Respiratory distress syndrome (RDS) - Need for/and duration of endotracheal mechanical ventilation (EMV) - Need for more than one dose of surfactant - Necrotizing enterocolitis (NEC) - Retinopathy of prematurity (ROP) - Sepsis - Length of stay in the neonatal intensive care unit (NICU) (days or weeks) - Seizures before NICU discharge - Neonatal encephalopathy or hypoxic ischemic encephalopathy (HIE) - Epilepsy - Neurodevelopmental impairment defined as one or more of cerebral palsy (CP) mental development impairment or psychomotor development impairment hearing deficit, visual deficit (at 1-2 years, 3-4 years, or after 5 years of age) - Any composite outcome measures reported by the authors and including our primary or secondary outcomes. |
| --- | --- |
| **Secondary maternal outcomes** | - Maternal mortality due to delivery complications - Admission to the intensive care unit (ICU) or high acuity unit - Post-partum hemorrhage - Need for transfusion - Deep venous thrombosis (DVT) - Pulmonary embolism (PE) - Type of caesarean incision (lower segment transverse, classic, “T” or “J”) - Caesarean section complications (e.g. caesarean hysterectomy, bowel injury bladder injury, wound infection, dehiscence or evisceration) - Type of anaesthesia (spinal, epidural, general) - Failed spinal or epidural analgesia, difficult or failed intubation - Perineal trauma (third or fourth degree lacerations or obstetrical anal sphincter injury) - Length of hospital stay - Any other relevant outcome or composite outcome measure encompassing our outcomes of interest reported by the authors |
