# Supplementary material for: What is the safest mode of delivery for extremely preterm cephalic/non-cephalic twin pairs? A systematic review and meta-analyses
Source: BMC Pregnancy Childbirth. 2017 Nov 29;17:397. doi: 10.1186/s12884-017-1554-7 (PMC5707900; doi:10.1186/s12884-017-1554-7)
Supplement: Supplementary file 3 — Summary table of excluded studies in a systematic review and meta-analyses on the safest mode of delivery for extremely preterm cephalic/non-cephalic twin pairs - author contacted but could not provide the necessary data or did not respond. (DOC 179 kb) [file 12884_2017_1554_MOESM3_ESM.doc]

# **Appendix 3** – Summary table of excluded studies in a systematic review and meta-analyses on the safest mode of delivery for extremely preterm cephalic/non-cephalic twin pairs - author contacted but could not provide the necessary data or did not respond

| **Study ID**  (Author,Year  Country  Study Period) | **Study Design** | **Reason for not including the study** |
| --- | --- | --- |
| **STUDIES FROM HIGH INCOME COUNTRIES**  **Author contacted**  **could not provide the necessary data or did not respond** | | |
| **Thomas, 2016**  Australia  1998-2009 | Retrospective cohort | - Outcome by mode of delivery not stratified by birth order: first and second twins breech analyzed together. - High order multiples included in analysis. |
| **Armson, 2006**  Canada  1988-2002 | Retrospective cohort | - Outcome by mode of delivery not stratified by gestational age <28 weeks nor by presentation of the twins. |
| **Smith, 2007**  England, Northern Ireland & Wales  1994-2003 | Retrospective cohort | - Outcome by mode of delivery not stratified by gestational age <28 weeks or by presentation. - Only discordant twin pairs for survival included in study. |
| **Sheay, 2004**  USA  1995-1997 | Retrospective cohort | - Outcome by mode of delivery not stratified by birth weight <1000g or gestational age <28 weeks nor by presentation. |
| **Garite, 2004**  USA  1997-2001 | Retrospective cohort | - Outcome by mode of delivery not stratified by presentation. |
| **Marttila, 2004**  Finland  1987-2000 | Retrospective cohort | - Outcome by mode of delivery not stratified by gestational age <28 weeks or by presentation. |
| **Vidovics, 2014**  Austria  2000-2009 | Retrospective cohort | - No twin <28 weeks in study (ascertained after author contact). |
| **Shinwell, 2004**  Israel  1995-1999 | Retrospective cohort | - Outcome by mode of delivery not stratified by gestational age <28 weeks nor by presentation. |
| **Kallen, 2015**  Sweden  2004-2007 | Prospective cohort | - Outcome by mode of delivery not provided separately for twins and singletons. |
| **Sentilhes, 2015**  France  1999-2010 | Retrospective cohort | - Outcome by planned mode of delivery not stratified by gestational age <28 weeks nor by presentation. |
| **Malloy, 2008**  USA  2000-2003 | Retrospective cohort | - Outcome by mode of delivery not provided separately for twins and singletons. |
| **Yang, 2005**  USA  1995-1997 | Retrospective cohort | - Outcome by mode of delivery not stratified by birth weight <1000g. |
| **Caukwell, 2002**  United Kingdom  1990-1997 | Retrospective cohort | - Outcome by mode of delivery not stratified by gestational age <28 weeks. |
| **Herbst, 2008**  Sweden  1980-2004 | Retrospective cohort | - Outcome by mode of delivery not stratified by gestational age <28 weeks. |
| **Winn, 2001**  USA  1994-1999 | Retrospective cohort | - Outcome by mode of delivery not stratified by gestational age <28 weeks. - Comparison of outcome by mode of delivery for first twins not presented in paper. |
| **Lodha, 2011**  Canada  2003-2007 | Retrospective cohort | - Outcome by mode of delivery not provided separately for twins and singletons. |
| **Dani, 2010**  Italy  1999-2007 | Prospective cohort | - Outcome by mode of delivery not stratified by presentation for twins. - High order multiples included in analysis. |
| **Hogberg, 2006**  Sweden  1997-2003 | Retrospective cohort | - Outcome by mode of delivery not provided separately for twins and singletons. |
| **Ginsberg, 2005**  USA  1997-2000 | Retrospective cohort | - Outcome by mode of delivery not stratified for birth weight <1000g. |
| **Markestad, 2005**  Norway  1999-2000 | Prospective cohort | - Outcome by mode of delivery not provided separately for twins and singletons. |
| **Jain, 2009**  USA  1997-2005 | Retrospective cohort | - Outcome by mode of delivery not provided separately for twins and singletons. |
| **Smith, 2002**  Scotland  1992-1997 | Retrospective cohort | - Outcome by mode of delivery not stratified by gestational age <28 weeks or by presentation. |
| **Rydhstroem, 2001**  Sweden  1991-1997 | Retrospective cohort | - Outcome by mode of delivery not stratified by gestational age less <28 weeks. |
| **Jaeger, 2004**  Germany  1985-1994 | Retrospective cohort | - Outcome by mode of delivery not provided separately for twins and singletons. |
| **STUDIES FROM LOW INCOME COUNTRIES**  **Author contacted**  **could not provide the necessary data or did not respond** | | |
| **Usta, 2005**  Lebanon  1984-2000 | Retrospective cohort | - Outcome by mode of delivery not stratified by gestational age <28weeks. |
| **Gupta, 2014**  India  2005 | Prospective cohort | - Only one twin pair of gestational age <28 weeks in sample (ascertained after author contact). |
| **Atis, 2011**  Turkey  2001-2007 | Retrospective cohort | - Outcome by mode of delivery not stratified by gestational age <28weeks. |
| **Aisien, 2000**  Nigeria  1992-1998 | Retrospective cohort | - Outcome by mode of delivery not stratified by gestational age <28weeks nor presentation or birth order. |
| **Usta, 2002**  Lebanon  1984-1994 | Retrospective cohort | - Outcome by mode of delivery not stratified by birth weight <1000g or gestational age <28weeks. |
| **Tadic, 2003**  Croatia  1997-2001 | Cohort study | - Outcome by mode of delivery not provided separately for twins and singletons. |
| **Sbeiti, 2005**  Lebanon  1991-2002 | Retrospective cohort | - Outcome by mode of delivery not provided separately for twins and singletons. No stratification by gestational age <28 weeks or birth weight <1000g. |
| **Ziadeh, 2000**  Jordan  1994-1999 | Cohort study | - Outcome by mode of delivery not stratified by birth weight <1000g or by gestational age <28weeks nor presentation nor by presentation and birth order at once. |
| **Piekarski, 1997**  Poland  1986-1995 | Retrospective cohort | - Outcome by mode of delivery not stratified by gestational age <28weeks. |
| 1. Thomas PE, Petersen SG, Gibbons K. The influence of mode of birth on neonatal survival and maternal outcomes at extreme prematurity: A retrospective cohort study. Aust N Z J Obstet Gynaecol. 2016;56:60-68.  2. Armson BA, O'Connell C, Persad V, Joseph KS, Young DC, Baskett TF. Determinants of perinatal mortality and serious neonatal morbidity in the second twin. Obstet Gynecol. 2006;108:556-564.  3. Smith GCS, Fleming KM, White IR. Birth order of twins and risk of perinatal death related to delivery in England, Northern Ireland, and Wales, 1994-2003: retrospective cohort study. BMJ. 2007;334:576-576.  4. Sheay W, Ananth CV, Kinzler WL. Perinatal mortality in first- and second-born twins in the United States. Obstet Gynecol. 2004;103:63-70.  5. Garite TJ, Clark RH, Elliott JP, Thorp JA. Twins and triplets: The effect of plurality and growth on neonatal outcome compared with singleton infants. Am J Obstet Gynecol. 2004;191:700-707.  6. Marttila R, Kaprio J, Hallman M. Respiratory distress syndrome in twin infants compared with singletons. Am J Obstet Gynecol. 2004;191:271-276.  7. Vidovics M, Jacobs VR, Fischer T, Maier B. Comparison of fetal outcome in premature vaginal or cesarean breech delivery at 24-37 gestational weeks. Archives of Gynecology and Obstetrics. 2014;290:271-281.  8. Shinwell ES, Blickstein I, Lusky A, Reichman B. Effect of birth order on neonatal morbidity and mortality among very low birthweight twins: a population based study. Arch Dis Child Fetal Neonatal Ed. 2004;89:F145-148.  9. Kallen K, Serenius F, Westgren M, Marsal K, Fritz T, Holmgren PA et al. Impact of obstetric factors on outcome of extremely preterm births in Sweden: Prospective population-based observational study (EXPRESS). Acta Obstet Gynecol Scand. 2015;94:1203-1214.  10. Sentilhes L, Oppenheimer A, Bouhours AC, Normand E, Haddad B, Descamps P et al. Neonatal outcome of very preterm twins: policy of planned vaginal or cesarean delivery. Am J Obstet Gynecol. 2015;213:73.e71-77.  11. Malloy MH. Impact of cesarean section on neonatal mortality rates among very preterm infants in the United States, 2000-2003. Pediatrics. 2008;122:285-292.  12. Yang Q, Wen SW, Chen Y, Krewski D, Fung Kee Fung K, Walker M. Neonatal death and morbidity in vertex-nonvertex second twins according to mode of delivery and birth weight. Am J Obstet Gynecol. 2005;192:840-847.  13. Caukwell S, Murphy DJ. The effect of mode of delivery and gestational age on neonatal outcome of the non-cephalic- presenting second twin. Am J Obstet Gynecol. 2002;187:1356-1361.  14. Herbst A, Kallen K. Influence of mode of delivery on neonatal mortality in the second twin, at and before term. BJOG. 2008;115:1512-1517.  15. Winn HN, Cimino J, Powers J, Roberts M, Holcomb W, Artal R et al. Intrapartum management of nonvertex second-born twins: a critical analysis. Am J Obstet Gynecol. 2001;185:1204-1208.  16. Lodha A, Zhu Q, Lee SK, Shah PS. Neonatal outcomes of preterm infants in breech presentation according to mode of birth in Canadian NICUs. Postgraduate medical journal. 2011;87:175-179.  17. Dani C, Poggi C, Bertini G, Pratesi S, Di Tommaso M, Scarselli G et al. Method of delivery and intraventricular haemorrhage in extremely preterm infants. J Matern Fetal Neonatal Med. 2010;23:1419-1423.  18. Hogberg U, Hakansson S, Serenius F, Holmgren PA. Extremely preterm cesarean delivery: a clinical study. Acta Obstet Gynecol Scand. 2006;85:1442-1447.  19. Ginsberg NA, Levine EM. Delivery of the second twin. International Journal of Gynecology & Obstetrics. 2005;91:217-220.  20. Markestad T, Kaaresen PI, Ronnestad A, Reigstad H, Lossius K, Medbo S et al. Early death, morbidity, and need of treatment among extremely premature infants. Pediatrics. 2005;115:1289-1298.  21. Jain NJ, Kruse LK, Demissie K, Khandelwal M. Impact of mode of delivery on neonatal complications: trends between 1997 and 2005. J Matern Fetal Neonatal Med. 2009;22:491-500.  22. Smith GC, Pell JP, Dobbie R. Birth order, gestational age, and risk of delivery related perinatal death in twins: retrospective cohort study. BMJ. 2002;325:1004.  23. Rydhstroem H. Should all twins be delivered by caesarean section? A preliminary report. Twin Res. 2001;4:156-158.  24. Jaeger M, Grussner SE, Omwandho CO, Klein K, Tinneberg HR, Klingmuller V. [Cranial sonography for newborn screening: a 10-year retrospective study in 11,887 newborns]. RoFo : Fortschritte auf dem Gebiete der Rontgenstrahlen und der Nuklearmedizin. 2004;176:852-858.  25. Usta IM, Rechdan JB, Khalil AM, Nassar AH. Mode of delivery for vertex-nonvertex twin gestations. International Journal of Gynecology and Obstetrics. 2005;88:9-14.  26. Gupta P, Faridi MM, Goel N, Zaidi ZH. Reappraisal of twinning: epidemiology and outcome in the early neonatal period. Singapore Med J. 2014;55:310-317.  27. Atis A, Aydin Y, Donmez M, Sermet H. Apgar scores in assessing morbidity of the second neonate of cephalic/non-cephalic twins in different delivery modes. J Obstet Gynaecol. 2011;31:43-47.  28. Aisien AO, Olarewaju RS, Imade GE. Twins in Jos Nigeria: a seven-year retrospective study. Med Sci Monit. 2000;6:945-950.  29. Usta IM, Nassar AH, Awwad JT, Nakad TI, Khalil AM, Karam KS. Comparison of the perinatal morbidity and mortality of the presenting twin and its co-twin. J Perinatol. 2002;22:391-396.  30. Tadic E, Stefanic-Mitrovic D, Milic N, Kulisic D, Baraka K. Should we increase the number of cases of Cesarean sections?. [Croatian]. Gynaecologia et Perinatologia. 2003;12:33-36.  31. Sbeiti N, Ziedeh F, Ramadan M, Lababidi H, Rajab M. Outcomes of premature and very-low-birth-weight infants from 1991 to 2002. Journal Medical Libanais. 2005;53:162-167.  32. Ziadeh SM, Badria LF. Effect of mode of delivery on neonatal outcome of twins with birthweight under 1500 g. Arch Gynecol Obstet. 2000;264:128-130.  33. Piekarski P, Czajkowski K, Maj K, Milewczyk P. [Neonatal outcome depending on the mode of delivery and fetal presentation in twin gestation]. Ginekol Pol. 1997;68:187-192. | | |
