# Supplementary material for: What is the safest mode of delivery for extremely preterm cephalic/non-cephalic twin pairs? A systematic review and meta-analyses
Source: BMC Pregnancy Childbirth. 2017 Nov 29;17:397. doi: 10.1186/s12884-017-1554-7 (PMC5707900; doi:10.1186/s12884-017-1554-7)
Supplement: Supplementary file 4 — GRADE assessment for the primary composite outcome (neonatal death and severe brain injury), neonatal death and respiratory distress syndrome in a systematic review and meta-analyses for the safest mode of delivery for extremely preterm cephalic/non-cephalic twin pairs. (DOC 61 kb) [file 12884_2017_1554_MOESM4_ESM.doc]

# **Appendix 4** – GRADE assessment for the primary composite outcome (neonatal death and severe brain injury), neonatal death and respiratory distress syndrome in a systematic review and meta-analyses for the safest mode of delivery extremely preterm cephalic/non-cephalic twin pairs

| **Quality assessment** | | | | | | | **№ of patients** | | **Effect** | | **Quality** |
| --- | --- | --- | --- | --- | --- | --- | --- | --- | --- | --- | --- |
| **№ of studies** | **Study design** | **Risk of bias** | **Inconsistency** | **Indirectness** | **Imprecision** | **Other considerations** | **caesarean section** | **vaginal delivery** | **Relative (95% CI)** | **Absolute (95% CI)** |
| **Neonatal death or SBI in survivors - First twin** | | | | | | | | | | | |
| 2 | observational studies | very serious ^a^ | serious ^b^ | not serious | serious ^c^ | all plausible residual confounding would suggest spurious effect, while no effect was observed | 1/6 (16.7%) | 4/8 (50.0%) | **OR 0.35** (0.00 to 92.61) | **241 fewer per 1,000** (from -- to 489 more) | ⨁◯◯◯ VERY LOW |
| **Neonatal death or SBI in survivors - Second twin** | | | | | | | | | | | |
| 2 | observational studies | very serious ^c^ | serious ^b^ | not serious | serious ^c^ | all plausible residual confounding would suggest spurious effect, while no effect was observed | 3/7 (42.9%) | 2/6 (33.3%) | **OR 1.69** (0.04 to 72.81) | **125 more per 1,000** (from 314 fewer to 640 more) | ⨁◯◯◯ VERY LOW |
| **Neonatal death or SBI in survivors – Both twins** | | | | | | | | | | | |
| 2 | observational studies | very serious ^a^ | serious ^b^ | not serious | serious ^c^ | all plausible residual confounding would suggest spurious effect, while no effect was observed | 4/13 (30.8%) | 6/14 (42.9%) | **OR 0.83** (0.05 to 13.43) | **45 fewer per 1,000** (from 392 fewer to 481 more) | ⨁◯◯◯ VERY LOW |
| **Neonatal death - First twin** | | | | | | | | | | | |
| 2 | observational studies | very serious ^a^ | not serious | not serious | serious ^c^ | all plausible residual confounding would suggest spurious effect, while no effect was observed | 0/7 (0.0%) | 2/10 (20.0%) | **OR 0.36** (0.03 to 4.40) | **117 fewer per 1,000** (from 193 fewer to 324 more) | ⨁◯◯◯ VERY LOW |
| **Neonatal death - Second twin** | | | | | | | | | | | |
| 2 | observational studies | very serious ^a^ | serious ^b^ | not serious | serious ^c^ | all plausible residual confounding would suggest spurious effect, while no effect was observed | 2/8 (25.0%) | 2/9 (22.2%) | **OR 1.31** (0.02 to 79.60) | **50 more per 1,000** (from 217 fewer to 736 more) | ⨁◯◯◯ VERY LOW |
| **Neonatal death – Both twins** | | | | | | | | | | | |
| 2 | observational studies | very serious ^a^ | not serious | not serious | serious ^c^ | all plausible residual confounding would suggest spurious effect, while no effect was observed | 2/15 (13.3%) | 4/19 (21.1%) | **OR 0.73** (0.10 to 5.46) | **48 fewer per 1,000** (from 185 fewer to 382 more) | ⨁◯◯◯ VERY LOW |
| **SBI in survivors - Fist twin** | | | | | | | | | | | |
| 2 | observational studies | very serious ^a^ | serious ^b^ | not serious | serious ^c^ | all plausible residual confounding would suggest spurious effect, while no effect was observed | 1/6 (16.7%) | 2/6 (33.3%) | **OR 0.59** (0.00 to 154.35) | **106 fewer per 1,000** (from -- to 654 more) | ⨁◯◯◯ VERY LOW |
| **SBI in survivors - Second twin** | | | | | | | | | | | |
| 2 | observational studies | very serious ^a^ | not serious | not serious | serious ^c^ | all plausible residual confounding would suggest spurious effect, while no effect was observed | 1/5 (20.0%) | 0/4 (0.0%) | **OR 1.00** (0.02 to 40.28) | **0 fewer per 1,000** (from 0 fewer to 0 fewer) | ⨁◯◯◯ VERY LOW |
| **SBI in survivors – Both twins** | | | | | | | | | | | |
| 2 | observational studies | very serious ^a^ | not serious | not serious | serious ^c^ | all plausible residual confounding would suggest spurious effect, while no effect was observed | 2/11 (18.2%) | 2/10 (20.0%) | **OR 0.76** (0.03 to 17.34) | **40 fewer per 1,000** (from 193 fewer to 613 more) | ⨁◯◯◯ VERY LOW |
| **RDS - First twin** | | | | | | | | | | | |
| 2 | observational studies | very serious ^a^ | not serious | not serious | serious ^c^ | all plausible residual confounding would suggest spurious effect, while no effect was observed | 13/14 (92.9%) | 15/15 (100.0%) | **OR 0.23** (0.01 to 6.25) | **0 fewer per 1,000** (from 0 fewer to 0 fewer) | ⨁◯◯◯ VERY LOW |
| **RDS - Second twin** | | | | | | | | | | | |
| 2 | observational studies | very serious ^a^ | not serious | not serious | serious ^c^ | all plausible residual confounding would suggest spurious effect, while no effect was observed | 13/14 (92.9%) | 13/15 (86.7%) | **OR 1.60** (0.12 to 20.99) | **46 more per 1,000** (from 126 more to 428 fewer) | ⨁◯◯◯ VERY LOW |
| **RDS – Both twins** | | | | | | | | | | | |
| 2 | observational studies | very serious ^a^ | not serious | not serious | serious ^c^ | all plausible residual confounding would suggest spurious effect, while no effect was observed | 26/28 (92.9%) | 28/30 (93.3%) | **OR 0.77** (0.10 to 5.87) | **18 fewer per 1,000** (from 55 more to 350 fewer) | ⨁◯◯◯ VERY LOW |

* Risk of bias was assessed as ‘very serious’ if the average Newcastle Ottawa Scale score for the included studies was <7 points.

†Imprecision was assessed as ‘serious’, since the 95% confidence intervals span no effect, and do not show appreciable benefit or exclude harm
